# Mechanisms of acquired resistance to fibroblast growth factor receptor targeted therapy

**DOI:** 10.20517/cdr.2019.42

**Published:** 2019-09-19

**Authors:** David K. Lau, Laura Jenkins, Andrew Weickhardt

**Affiliations:** ^1^Olivia Newton John Cancer Research Institute, Heidelberg, Victoria 3084, Australia.; ^2^School of Cancer Medicine, La Trobe University, Heidelberg, Victoria 3084, Australia.; ^3^Department of Medical Oncology, Austin Health, Heidelberg, Victoria 3084, Australia.

**Keywords:** Fibroblast growth factor receptor, targeted therapy, acquired resistance

## Abstract

Oncogenic activation of the fibroblast growth factor receptor (FGFR) through mutations and fusions of the *FGFR* gene occur in a variety of different malignancies such as urothelial carcinoma and cholangiocarcinoma. Inhibition of the kinase domain of the FGFR with targeted oral FGFR inhibitors has been shown in both preclinical and early phase clinical trials to lead to meaningful reductions in tumour size and larger confirmatory randomized trials are underway. Acquired resistance to FGFR inhibition using a variety of mechanisms that includes, activation of alternate signaling pathways and expansion of tumour clones with gatekeeper mutations in the *FGFR* gene. This review summarizes the acquired resistance mechanisms to FGFR therapy and therapeutic approaches to circumventing resistance.

## Introduction

The fibroblast growth factor receptor (FGFR) is a membrane bound protein that regulates cellular functions including cell proliferation, cell survival, differentiation and migration^[1]^. Activation of the FGFR family (FGFR1, FGFR2, FGFR3 and FGFR4) leads to increased downstream activation of oncogenic pathways such as MAPK and AKT^[2]^. Amplifications, mutations and aberrant fusions of the *FGFR* gene lead to constitutively activated downstream signaling of these pathways with enhanced cellular growth and migration^[3]^.

Cancers such as breast, lung, gastric, urothelial and intrahepatic cholangiocarcinoma harbor hyperactivation of FGFR signaling pathways due to oncogenic aberrations of FGFR family members, although the nature of the oncogenic alteration is different between each cancer type.

Recently, a wide variety of orally available targeted pan-FGFR inhibitors such as derazantinib (ARQ-087, Arqule)^[4]^, AZD4547 (AstraZeneca)^[5]^, infigratinib (BGJ398, Novartis)^[6]^, erdafitinib (JNJ-42756493, Janssen)^[7]^, TAS-120 (Taiho)^[8]^ and pemigatinib (INCB054828, InCyte)^[9]^ with specificity for inhibition of the kinase domain of the activated FGFR protein have evolved from preclinical testing to early phase clinical trials. Anti-tumour activity in clinical trials of urothelial carcinoma and cholangiocarcinoma has led to larger confirmatory clinical trials that may lead to registration of these agents.

Preclinical trials and early phase testing have demonstrated resistance occurs to these targeted agents and similar phenomenon is seen with other kinase inhibitors, such as EGFR^[10]^, ROS1^[11]^ and cKIT inhibitors^[12]^. The goal of this review is to outline the outcomes of preclinical and clinical studies of acquired resistance to selective FGFR inhibition and development of rational therapeutic strategies to circumvent resistance.

## Activating FGFR alterations in cancer

Identification of *FGFR* gene fusions, mutations and amplifications in a range of cancer types has driven the study of FGFR inhibitors in these tissue and biomarker selected populations. Gene fusions involving *FGFR2* occur in 7%-14% of intrahepatic cholangiocarcinomas. The first reported constitutively active fusion gene was the *FGFR2-BICC1*^[13]^. Other reported fusion partners of *FGFR2* include *CCDC6*^[14]^, *PPHLN1*^[15]^, *AHCYL1*^[16]^, *TXLNA1* and *KCTD1*^[17]^. The breakpoint in nearly all *FGFR2* fusions is located in exon 18, distal to the kinase domain of the FGFR receptor. In non-muscle invasive bladder cancer, *FGFR3* mutations can be detected in 50%-60% cases. By contrast, the prevalence of *FGFR3* mutations is significantly lower in muscle invasive bladder cancer (10%-15%)^[18,19]^. In addition, *FGFR3* fusion rearrangements are present in 6% of muscle invasive bladder cancer^[20]^. *FGFR2* mutations are present in ~10% of endometrial cancers^[21]^. The results of early phase trials of FGFR inhibitors in urothelial carcinoma (*FGFR3* mutations/translocations) and cholangiocarcinoma (*FGFR2* fusions) are shown in Table 1.

**Table 1:** Key clinical studies of selective pan-FGFR inhibitors in advanced cholangiocarcinoma and urothelial carcinoma

Study (author/year)	Drug	Phase, patients	Median PFS (95%CI)	Median OS (95%CI)	ORR
Cholangiocarcinoma					
Javle *et al*.^[33]^, 2018	Infigratinib	Phase II single arm *FGFR2* fusion (78.7%) *n* = 67	5.8 months (4.3-7.6)	NR	14.8% (9/61)
Mazzaferro *et al*.^[34]^, 2019	Derazantinib	Phase II single arm *FGFR2* fusion *n* = 29	5.7 months (4.04-9.2)	NR	20.7% (6/29)
Meric-Bernstam *et al*.^[37]^, 2018	TAS-120	Phase I dose escalation *FGFR2* fusions *n* = 28 Other FGFR alterations	NR	NR	*FGFR2* fusions 25% (7/28) Other *FGFR* alterations 18% (3/17)
Hollebecque *et al*.^[9]^, 2018	Pemigatinib	Phase II Cohort A: *FGFR2* fusion (*n* = 47) Cohort B: *FGFR*/*FGF* alterations (*n* = 22) Cohort C: no *FGFR* alteration *n* = 18	A: 6.8 months (3.6-9.2) B: 1.5 months C: 1.4 months	NR	A: 18% (8/45) B: 0% (0/22) C: 0% (0/18)
Chen *et al*.^[36]^, 2018	Erdafitinib	Asian cohort *FGFR2* fusions/ mutations *n* = 12	NR	NR	45.5% (5/11)
Urothelial carcinoma					
Pal *et al*.^[39]^, 2018	Infigratinib	Phase II single arm *FGFR3* fusion *n* = 67	3.75 months (3.09-5.39)	7.75 months (5.65-11.60)	25.4% (17/67)
Loriot *et al*.^[38]^, 2018	Erdafitinib	Phase II, single arm *FGFR2*/*FGFR3* fusions/mutations *n* = 96	5.5 months	13.8 months	40%
Joerger *et al*.^[86]^, 2018	Rogaratinib	Phase I expansion *FGFR3* mRNA alterations/high expression (*n* = 52)	NR	NR	24% (12/51)
Necchi *et al*.^[87]^, 2018	Pemigatinib	Phase II Cohort A: *FGFR3* mutations/fusions (*n* = 64) Cohort B: Other *FGFR*/*FGF* alterations (*n* = 36)	NR	NR	A: 25% (13/51) B:3% (1/36)

PFS: progression free survival; OS: overall survival; CI: confidence interval; ORR: objective response rate; NR: not reported

*FGFR1* amplifications are present in several tumour types including squamous non-small cell lung (10%-20%)^[22,23]^, hormone positive breast (10%), head and neck (10%-17%), squamous oesophageal (20%) and ovarian cancer (9%)^[24]^. *FGFR2* amplifications can be detected in triple negative breast (4%)^[25]^ and gastric cancer (4%-7%)^[26]^. Low frequency FGFR alterations have been observed in sarcomas, glioma, pancreatic, renal, colorectal neuroendocrine cancers^[24]^. Despite strong preclinical rationale^[27-29]^, FGFR inhibitors in molecularly selected subgroups of squamous cell lung cancer^[30]^, breast cancer^[31]^ and gastric carcinoma^[32]^ to date have not shown significant efficacy, potentially due to the inclusion of tumour types defined by *FGFR* amplification, rather than mutations or fusions, which are more likely to respond to FGFR inhibition. The clinical evidence for targeting these tumours types and its respective *FGFR* genetic aberration are summarised in this section.

## Clinical trials in cholangiocarcinoma

In a proof of concept phase II study of infigratinib in advanced or metastatic cholangiocarcinoma with *FGFR2* aberrations (*n* = 61), the overall response rate (ORR) was 14.8%. Responses were observed only in patients with *FGFR2* fusions (*n* = 48) where the ORR was 18.8%. The median overall survival (OS) was 5.8 months (95%CI: 4.3-7.6 months)^[33]^. Similar results were observed with the FGFR inhibitor derazantinib (ARQ-087). In a phase I/II open label study in patients with *FGFR2* fusion cholangiocarcinoma (*n* = 29), the ORR was 20.7% and disease control rate (DCR) was 82.8%. Estimated median progression free survival was 5.7 months (95%CI: 4.04-9.2 months)^[34]^. An expanded cohort using the 300 mg dose is currently underway (NCT03230318). In a phase I dose expansion cohort of *FGFR2* translocated cholangiocarcinoma receiving erdafinitib (*n* = 11), objective responses were observed in 3 patients (27.3%) with an additional 3 patients achieving stable disease^[35]^. Preliminary results of erdafitinib in a phase II study in an Asian cohort reported an ORR of 45%^[36]^. Clinical activity has been reported in non-randomised studies of *FGFR2* translocated cholangiocarcinoma with TAS-120 and pemigatinib^[9,37]^. A phase III study comparing infigratinib and chemotherapy (cisplatin and gemcitabine) in *FGFR2* fusion cholangiocarcinoma in the first-line setting is currently underway (NCT03773302).

## Clinical trials in urothelial carcinoma

In the BLC2001 phase II study of metastatic urothelial carcinoma with *FGFR* alterations, 96 patients who received at least one line of chemotherapy or were chemotherapy naïve and ineligible to receive cisplatin were enrolled to receive erdafitinib 8 mg/d in 28-day cycles with doses increased to 9 mg/d based on serum phosphorus (phosphate) levels. The ORR was 42% (3% complete response, 39% partial response). The progression free survival (PFS) and OS were 5.5 and 13.8 months respectively^[38]^. Based upon these results, erdafitinib was granted breakthrough therapy designation by the Food and Drug Administration. In a phase II trial, among 67 patients with *FGFR3* mutations/fusion positive bladder cancer enrolled to receive infigratinib, objective responses were observed in 25.4% of subjects. However, responses were not sustained with median PFS of 3.75 months (95%CI: 3.09-5.39 months)^[39]^. A randomised phase III trial comparing the efficacy of erdafitinib against chemotherapy or pembrolizumab is underway (NCT03390504). A trial in the adjuvant setting with pemigatinib is also recruiting (NCT03656536).

## Clinical trials in gastric cancer

In contrast to the trials of FGFR inhibitors in cholangiocarcinoma and urothelial cancer, results from gastric cancer studies have been disappointing. Despite preclinical evidence of *FGFR* amplification corresponding with sensitivity to this drug class, results from treating patients with tumours harbouring *FGFR* amplification have been disappointing. In a phase I expanded cohort study of AZD4547, in 13 patients with FGFR amplified gastric cancer one patient had a partial response. Of note, the partial responder had clusters of *FGFR1/2* gene amplification^[40]^. In a phase II translational clinical trial, 3 of the 9 patients with *FGFR2* amplified GC achieved an objective response to AZD4547 patients with homogenous, high level *FGFR2* amplification (FISH ratio > 5) were most likely to respond^[41]^. In a phase I study of LY2874455 in patients with advanced-stage solid cancers, there were two partial responses (4%, 2/51) seen in patients with *FGFR2* non-amplified gastric cancer^[42]^.

In the SHINE study, a randomised phase II open label study of metastatic gastric cancer, 71 patients with *FGFR2* amplification or polysomy were randomised to receive AZD4547 (amplified *n* = 18, polysomy *n* = 20; total *n* = 41) or paclitaxel (amplified *n* = 15, polysomy *n* = 15, *n* = 30). The median PFS was 1.8 months in the AZD4547 arm and 3.5 months in the paclitaxel arm (HR 1.57, 80%CI: 1.12-2.21). The ORR was 2.6% in the AZD4547 arm and 23.3% in the paclitaxel arm. The median OS was 5.5 and 6.6 months for the AZD4547 and paclitaxel arms, respectively (HR 1.31, 80%CI: 0.89-1.95)^[32]^. Further work is required to identify why most patients with *FGFR* amplification have *de novo* resistance to this drug class and represents opportunities for clinical translational research and development of alternate strategies such as FGFR selective antibodies^[43]^.

## Clinical trials in squamous lung cancer

In a phase Ib study in *FGFR1* amplified squamous cell lung cancer, 15 patients received AZD4547. There was one partial response (8%, 1/13)^[44]^ and the median overall survival was 4.9 months^[44]^. In a phase I study of infigratinib, amongst the 27 patients with squamous cell lung cancer harbouring a *FGFR1* amplification, there were 4 partial responses (8%)^[31]^. In the phase II LungMAP umbrella trial, there was only one objective response amongst 25 patients with evaluable disease with *FGFR* amplified or mutated squamous cell lung cancer^[30]^.

## Clinical trials in breast cancer

In a phase II cohort of patients with HER2-negative breast cancer with *FGFR1* amplification, 8 patients received AZD4547. There was one partial response (13%, 1/8)^[41]^. In a phase I study of erdafitinib, none of the six patients with *FGFR1* amplification achieved an objective response^[45]^. Similarly, no objective responses (0/31) were observed in a phase I study of infigratinib in breast cancer (*FGFR1/2*, amplified *n* = 25)^[31]^. The lack of observed efficacy may be due to inadequate patient selection, ineffective compounds or the lack of oncogenic addiction of tumors harboring *FGFR1* amplifications. Co-amplification of other genes within the *FGFR1* containing 8p11-12 locus such as *ZNF708* may act as an oncogenic driver^[46]^. *FGFR1* amplifications are also associated with amplification of the 11q13 locus which contains putative oncogenes such as *CCND1* and *FGF19*^[47]^.

## Clinical trials in other tumour types

In a phase II basket trial recruiting patients with *FGFR* and *FGF* ligand alterations, responses were observed in ovarian (*n* = 2), head and neck (*n* = 4), and primary CNS cancer (*n* = 1) with infigratinib^[48]^. In a phase I study of erdafinitib, responses were observed in a patient with endometrial cancer (*FGFR2* fusion) and glioblastoma (*FGFR3* fusion)^[45]^. These studies suggest that basket studies that recruit patients based on molecular characterisation represent a potential avenue to demonstration of drug activity and registration, especially in the context of the increasing use of next generation gene sequencing and profiling.

## Mechanisms of acquired resistance

Many pre-clinical studies have been conducted and are in progress to identify mechanisms of resistance to FGFR inhibitors that may contribute to poor clinical trial performance. Most of this work has been performed on urothelial, lung and gastric cancer cell lines which may not accurately reflect mechanisms of resistance in other tumour types (i.e., cholangiocarcinoma). Additionally these *in vitro* cell lines have a high degree of clonality and may not recapitulate the heterogeneity of human disease. Many of these mechanisms have not been validated in the clinical arena, highlighting the importance of post-progression tumour sampling and other novel strategies for detecting resistance mechanisms, such as plasma ctDNA monitoring for emergent mutations. Urine cfDNA is another promising modality for liquid biopsy in urothelial carcinoma^[49]^.

Despite limitations these pre-clinical studies involving models FGFR aberrations have demonstrated multiple mechanisms of acquired resistance to FGFR inhibitors which will be reviewed here. These include bypass signaling, epithelial to mesenchymal transition (EMT) and the emergence of secondary mutations in FGFR known as gatekeeper mutations. These studies have identified potential therapeutic strategies that can enhance the modest clinical benefit of FGFR targeting to date.

## Activation of alternate receptor tyrosine kinases

**Figure 1:**
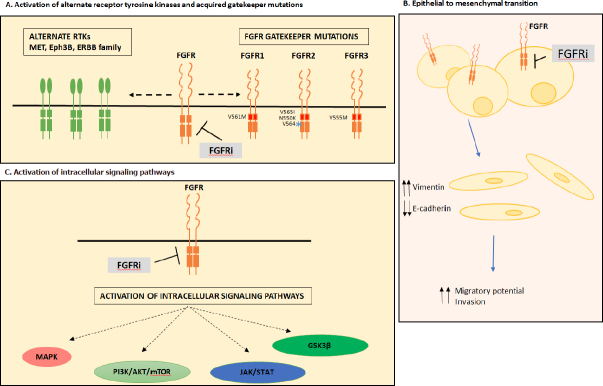
Mechanisms of acquired resistance to FGFR inhibition. Mechanisms of acquired resistance to FGFR inhibition can occur through activation of alternate receptor tyrosine kinases and acquired gatekeeper mutations (A), epithelial to mesenchymal transition (B) or activation of intracellular signaling pathways (C). FGFR: fibroblast growth factor receptor; FGFRi: fibroblast growth factor receptor inhibitor; RTK: receptor tyrosine kinase; MAPK: mitogen activation protein kinase; JAK/STAT: Janus kinase/signal transducers and activators of transcription; GSK3β: glycogen synthase kinase 3 beta

Resistance through bypass signaling occurs through the loss or switch of dependence of FGFR to other receptor tyrosine kinases, such as MET^[50,51]^, Eph3B^[52]^, ERBB2/3^[53]^ or EGFR^[29]^ [Figure 1].

Upregulation of the receptor tyrosine kinase MET has been described in DMS114 lung cancer cells (*FGFR1* amplified), made resistant to infigratinib^[50]^ and H1581 lung cancer cells (*FGFR1* amplified) made resistant to AZD4547 and the FGFR inhibitor rogaratinib^[51]^. In DMS114 resistant cells, transcriptional upregulation of MET led to reactivation of the MAPK signaling pathway. Similarly, in H1581 resistant cells, the elevated levels of MET led to increased downstream signaling pathways which were found to activate signaling in a ERBB3 dependent manner in cells resistant to AZD4547 and an ERBB3 independent manner in cells resistant to BAY1163877^[51]^. The increased phosphorylation of the Ephrin 3B (Eph3B) receptor was found to be associated with acquired FGFR resistance in SNU-16 (*FGFR2* amplified) gastric cancer cells, which could be reversed with small molecule inhibitors of Eph3B^[52]^.

Switch to ERBB3 dependency has been further demonstrated in RT112 urothelial cancer cells (*FGFR3*-*TACC3* fusion) following chronic exposure to infigratinib through upregulation of ERBB2/3 ligands^[53]^. Interestingly, upregulation of ERBB ligands only mediated resistance in FGFR3 dependent cells and not FGFR1 or FGFR2 dependent lines suggesting mechanisms of resistance may differ according to the FGFR alteration and cancer type^[53]^. EGFR has been shown to be a mediator of both acute and acquired resistance in *FGFR3* mutant or fusion cell lines^[29]^. The sensitivity of FGFR inhibitors in FGFR3 dysregulated cell lines is largely mediated by intrinsic activation of EGFR. Acquired resistance in FGFR3 dependent cell lines occurs as EGFR is upregulated upon inhibition of FGFR through the release of negative feedback mechanisms partially compensating for the loss of FGFR signaling. Alternatively, intrinsic resistance to FGFR inhibition in *FGFR3* mutant cells is due to EGFR dependency despite the presence of *FGFR3* activating mutations, whereby EGFR is able to repress FGFR3 expression^[29]^.

Using a kinome-wide CRISPR/Cas9 screen, 20 kinases involving ILK (Integrin-linked kinase), SRC, and EGFR signaling were found to alter sensitivity to FGFR inhibition in *FGFR2* amplified gastric cancer cell lines. Furthermore, in FGFR2^[54]^ and FGFR3^[29]^ dependent cell lines, co-targeting the FGFR inhibitor and EGFR or ERBB2/3 enhances anti-proliferative effects.

## Activation of intracellular signaling pathways

Bypass signaling can also occur due to changes within the PI3K/AKT/mTOR^[55-57]^, MAPK^[50,58,59]^, STAT3^[60]^ and GSK3β^[61]^ signaling pathways. Increased PI3K/AKT/mTOR signaling, independent of changes in upstream receptor tyrosine kinases has been described in DMS114 lung cancer cells (*FGFR1* amplified) and RT112 urothelial cancer cells (*FGFR3*-*TACC3*) following chronic treatment with infigratinib^[56]^. Using deep sequencing, the dependency of PI3K/AKT/mTOR signaling in resistant DMS114 cells were found to be mediated by the emergence of an *AKT1* mutation^[57]^. In murine cell lines of stem cell leukemia syndrome containing *FGFR1* amplification and made resistant to the non-selective FGFR inhibitor ponatinib, resistance was mediated by increased PI3K/AKT/mTOR signaling due to mutational inactivation of *PTEN*, a negative regulator of the pathway^[55]^.

Reactivation of PI3K/AKT/mTOR also occurs by of Pleckstrin Homology-Like Domain, family A, member 1 (PLHDA1) expression, a negative downregulator of PIP_3_/AKT binding. This effect has been observed in FGFR2 driven endometrial cancer cell lines with acquired resistance to FGFR inhibition and *ERBB2* amplified breast cancer cells treated with anti-HER2 therapy suggested PLHDA1 may be a common resistance mechanism in RTK driven cancers^[62]^. Synergistic anti-tumour responses have been observed by co-targeting the FGFR and PI3K/AKT/mTOR pathway with PI3K^[63]^, mTOR^[64,65]^ and Akt inhibitors^[56,57,66]^. The latter combination may be more effective in tumours harbouring a *PIK3CA* or *PIK3R1* mutation^[66]^.

Constitutive activation of the MAPK signaling pathway has been shown to mediate FGFR resistance in both *FGFR1* and *FGFR2* amplified cell lines, however through different mechanisms. MAPK activation in *FGFR1* amplified lines is shown to be mediated by a secondary mutation in *NRAS*^[59]^ and through chromosomal arm loss on chromosome 12p resulting in downregulation of DUSP6^[50]^ a negative regulator of the MAPK pathway. Alternatively, in *FGFR2* amplified cells constitutive MAPK signaling was mediated through the emergence of the *BRAF* fusion kinase *JHM1D-BRAF* which is demonstrated to enhance the dimerization capacity of BRAF^[58]^.

STAT3 has also been implicated in mediating resistance to AZD4547 and infigratinib in H1581 lung cancer cells (*FGFR1* amplified) following induction of cognate receptors by the secretome. Acquired resistance was found to be overcome Hsp90 and HDAC inhibitors^[60]^. Independent phosphorylation and inactivation of GSK3β has been demonstrated as a mechanism of resistance to PDX models of *FGFR2* amplified diffuse gastric cancer made resistant through chronic treatment with AZD4547^[61]^. Co-targetting of FGFR and ILK, an upstream receptor of GSK3β in *FGFR2* amplified gastric cancer cell lines, resulted in synergistic anti-tumour responses^[54]^.

## Epithelial-mesenchymal transition

The emergence of EMT following chronic exposure to FGFR inhibitors has also been described as a mechanism of resistance^[67]^. Morphological changes defined by cells becoming more spindle shaped were observed in the gastric cancer cell line SNU-16 (*FGFR2* amplification), following chronic exposure to AZD4547, infigratinib or PD173074. Consistent with changes in EMT, resistant cells displayed upregulation of vimentin and downregulation of the epithelial marker E-cadherin^[52,67]^. Furthermore, morphological changes in FGFR inhibitor resistant cell lines have also been reported in multiple cell lines including; RT112 resistant urothelial (*FGFR3-TACC3*)^[53,56]^ and H1581 lung (*FGFR1* amplified)^[51]^. In addition to morphological changes, H1581 resistant cells also showed enhanced migratory potential and invasion through matrigel, consistent with an EMT phenotype^[53]^.

## Gatekeeper mutations

Gatekeeper mutations, that modify the binding pocket to prevent drug binding, including *FGFR1* V561M^[55,68,69]^, *FGFR2* V565I/N550K/V564^[70,71]^ and *FGFR3* V555M^[72]^ are a distinct mechanism of resistance to FGFR inhibitors. Importantly, this mechanism implies tumours maintain their dependence on FGFR signaling and downstream pathway activation^[73]^. The *FGFR1* V561 gatekeeper mutation is able to confer resistance though the stabilisation of the hydrophobic spine that favours the active conformation of FGFR1, which increases its autophosphorylation capacity^[69]^. Interestingly, the V561 mutation was shown to decrease the binding affinity to FGFR-VEGF inhibitor lucitanib by 500 fold whilst largely retaining binding affinity to the pan-FGFR inhibitor AZD4547^[69]^. Resistance to AZD4547 by the V561 mutation is mediated by increased phosphorylation of STAT3^[68]^. *FGFR2* gatekeeper mutations V565I and N550K have been verified in BaF3 cells overexpressing FGFR2 and made resistant to dovitinib with both mutations working through different molecular mechanisms^[70]^. The V565I mutation confers resistance through steric hindrance of the drug into the ATP binding cleft whereas the N550K mutation confers resistance through stabilisation of FGFR2 into the active conformation^[70]^. The *FGFR3* V555M mutation was identified in KMS-11 multiple myeloma cells (*FGFR3* Y373C) made resistant to the FGFR inhibitor AZ12908010^[72]^. Modelling of This mutation suggests amino acid substitution of valine to the methionine (with a larger side chain) would restrict access to the cavity adjacent to the ATP binding region^[72]^.

Using serial analysis of plasma cell-free DNA (cfDNA)^[74]^ isolated from three patients with *FGFR2* fusion positive cholangiocarcinoma, receiving infigratinib, Goyal *et al*.^[71]^ detected the emergence of resistance gatekeeper conferring mutations in *FGFR2*. Mutations at V564F, within the kinase domain developed in all three patients, suggesting this is common mechanism of resistance. Structural modelling suggested that this mutation caused a steric clash with infigratinib in the FGFR2 binding pocket. In two patients, multiple point mutations were detected (including V564F, N549H N549K, E565A, K659M, L617V) which are predicted to confer FGFR resistance. Conversely, PI3K pathway mutations were also detected in post progression and autopsy biopsies confirming the heterogeneity of these mutations underpins the complexity in treating these patients^[71]^. In urothelial carcinoma, the gatekeeper mutations *FGFR3* V443L, V443M, and L496V, were detected in cfDNA in 3 of 50 patients receiving erdafitnib^[39]^. Whilst not a gatekeeper mutation, a case report of a patient with *FGFR2* amplified gastric cancer who responded to LY2874455, reported a resistance conferring *FGFR2-ASCL5* fusion gene on post-progression biopsy^[75]^.

## Strategies to overcome acquired FGFR resistance

### Novel FGFR therapies

Development of covalently binding specific FGFR inhibitors an active area of investigation as a therapeutic strategy to overcome resistance facilitated by gatekeeper mutations in the ATP binding pocket of FGFR^[76]^. Importantly, this strategy has potential to increase the duration of response as observed in *EGFR*-mutated lung cancer^[77]^ and represents an opportunity for development structurally optimised inhibitors. The FGFR inhibitor UPR1376, a chloroacetamide derivative has demonstrated preclinical anti-tumour activity in *FGFR1* amplified lung cancer cell lines with acquired resistance to infigratinib^[78]^. Preliminary clinical results showed acquired resistance to infigratinib or Debio 1347 could be overcome with the covalently binding FGFR inhibitor TAS-120 in four patients with gatekeeper mutations^[73]^.

Monoclonal antibodies targeting FGFR have the ability to exert anti-tumour effects through antibody-dependent cell-mediated cytotoxicity^[79]^. Bemarituzumab (FPA144, Five Prime Therapeutics) targeting the FGFR2b isoform is currently in clinical testing (NCT03694522) in gastric carcinoma. FGF traps which sequester FGF ligands and prevent binding of ligands to FGFRs have been proposed as a therapeutic strategy, however its utility in *FGFR* mutational driven tumours is uncertain^[80]^. GSK3052230, a novel engineered FGF trap comprised of the extracellular domain of FGFR1 fused to the Fc portion and is in clinical testing in lung carcinoma (NCT01868022).

### Combination strategies

Clinical trials are exploring the strategy of upfront combination therapy to forestall the development of acquired resistance to selective FGFR inhibitors. In a phase I trial combination therapies of FGFR and PI3K inhibitors (infigratinib/BYL719) were tested in tumours with *PIK3CA* mutations or genetic alterations in *FGFR1-3*. Objective responses were observed in urothelial, head and neck, melanoma and anal cancer, however it is unclear whether this this combination is any more effective over single agent therapy^[81]^.

The combination of FGFR and immune checkpoint inhibitors are supported by marked anti-tumour activity in mouse models^[82]^. As checkpoint inhibition has been proven to be efficacious in refractory urothelial carcinoma^[83-85]^, the monoclonal anti-FGFR3 inhibitor B-701 is currently in clinical testing with the PD-1 antibody pembrolizumab (NCT03123055). Other immune checkpoint combinations in clinical testing in urothelial carcinoma include erdafitinib with JNJ-63723283 (NCT03473743), rogaratinib with atezolizumab (NCT03473756) and AZD4547 with durvalumab (NCT02546661).

## Conclusion

The mechanisms of FGFR resistance are diverse and include the activation of alternate receptor tyrosine kinases, induction of alternate cellular signaling pathways, induction of EMT and emergence of gatekeeper mutations. Further preclinical and translational clinical studies are paramount to define the mechanisms of resistance and design more rational treatments to overcome drug resistance. Combination treatment strategies to overcome bypass signaling and next generation FGFR inhibitors to circumvent gatekeeper mutations are promising avenues to improve the clinical use of FGFR inhibitors.

## Declarations

### Copyright

© The Author(s) 2019.
